# Supplementary material for: Global-scale prevalence of low nutrient use efficiency across major crops
Source: Nat Commun. 2025 Dec 10;16:11036. doi: 10.1038/s41467-025-66019-w (PMC12695879; doi:10.1038/s41467-025-66019-w)
Supplement: Supplementary file 1 — Supplementary Information [file 41467_2025_66019_MOESM1_ESM.pdf]

Supplementary information for "Global-scale prevalence of low nutrient use efficiency across major crops"

Ji Liu[1,2,3,4], Hai Wang[2], Josep Penuelas[3,4], Juan Mou[2], Manuel Delgado-Baquerizo[5], Jordi Sardans[3,4], Fernando Coello[3,4], Zhi Quan[6, 7], Tianyi Qiu[8], Yanyan Li[9], Yahui Guo[2], Ziying Hu[8], Yanrui Ying[8], Jingyi Lv[8], Yufeng Zhang[8], Wenfeng Tan[10], Guiyao Zhou[5], Lu-Jun Li[11], Linchuan Fang[8*]

[1]State Key Laboratory of Loess Sciences & National Observation and Research Station of Earth Critical Zone on the Loess Plateau, Institute of Earth Environment, Chinese Academy of Sciences, Xi'an, 710061, China
[2]Hubei Province Key Laboratory for Geographical Process Analysis and Simulation, Central China Normal University, Wuhan, 430079, China
[3]CSIC, Global Ecology Unit CREAF-CSIC-UAB, 08193 Bellaterra, Catalonia, Spain.
[4]CREAF, 08193 Cerdanyola del Vallès, 08193 Bellaterra, Catalonia, Spain
[5]Laboratorio de Biodiversidad y Funcionamiento Ecosistémico. Instituto de Recursos Naturales y Agrobiología de Sevilla (IRNAS). Consejo Superior de Investigaciones Científicas (CSIC). Av. Reina Mercedes 10, E-41012 Sevilla, Spain.
[6]Institute of Applied Ecology, Chinese Academy of Sciences, Shenyang, 110016, China
[7]Weifang Institute of Modern Agriculture and Ecological Environment, Weifang, 261041, China
[8]Key Laboratory of Green Utilization of Critical Non-metallic Mineral Resources, Ministry of Education, Wuhan University of Technology, Wuhan 430070, China
[9]College of Ecology and Environment, Xinjiang University, Urumqi, Xinjiang 830017, China
[10]College of Resources and Environment, Huazhong Agricultural University, Wuhan, China
[11]State Key Laboratory of Black Soils Conservation and Utilization, Northeast Institute of Geography and Agroecology, Chinese Academy of Sciences, Harbin 150081, PR China

***Corresponding author**
Dr. Linchuan Fang
Telephone: +86 1524920446
Key Laboratory of Green Utilization of Critical Non-metallic Mineral Resources, Ministry of Education, Wuhan University of Technology, Wuhan 430070, China
Email: flinc629@hotmail.com

Content

Supplementary Table 1 Comparison framework for nitrogen use efficiency (NUE) and phosphorus use efficiency (PUE) calculation approaches. 3
Supplementary Table 2 The global mean data of crop nitrogen (N) and phosphorus (P) content[1,2], crop residue-grain ratio[3] of crops of rice, wheat, maize, soybean and overall crop. 4
Supplementary Table 3 List of data used for calculating nitrogen from soil and phosphorus from soil....................................5
Supplementary Table 4 List of global maps used for extracting missing values and scaling up. 6
Supplementary Fig. 1 Trends in global harvested area, grain yield, input N(P) loads and input N(P) / Yield over time....................................7
Supplementary Fig. 2 Research period (a) and workflow (b) on global inorganic fertilizer nitrogen use efficiency (NUE) and phosphorus use efficiency (PUE) trends and patterns. 8
Supplementary Fig. 3 Nitrogen (N), phosphorus (P) fertilizer input intensities (a), and nitrogen (NUE), phosphorus use efficiency (PUE) (b) for major countries on temporal scales. 10
Supplementary Fig. 4 Nitrogen (NUE) and phosphorus (PUE) use efficiencies for rice (a), wheat (b), maize (c), soybean (d) in different climatic zones, major countries or regions, and globally. 12
Supplementary Fig. 5 Global-scale synergistic effects of nitrogen (NUE) and phosphorus use efficiency (PUE) of four major crops: rice, wheat, maize and soybean. 14
Supplementary Fig. 6 Temporal trends of nitrogen (NUE) and phosphorus use efficiency (PUE) of four major crops (rice, wheat, maize, soybean) under SSP126 (sustainable development-low forcing), SSP245 (moderate development-medium forcing), and SSP585 (fossil-fueled development-high forcing) scenarios. 15
Supplementary Fig. 7 The Preferred Reporting Items for Systematic Reviews and Meta-Analyses (PRISMA) for the meta-analysis 16
Supplementary Fig. 8 Prediction of weighted effect values of nitrogen (a) and phosphorus (b) from soil of the four major crops (rice, wheat, maize, soybean) and all crops by random forest models. 17
Supplementary Fig. 9 Nitrogen (NUE) and phosphorus use efficiency (PUE) and the proportion of nitrogen (nitrogen from soil) and phosphorus (phosphorus from soil) uptake from the soil for four major crops at different fertilizer input intensities. 19
Supplementary Fig. 10 The significance test of difference in rice, wheat, maize, soybean and all crops across years before and after their respective breakpoints. 20
Supplementary Fig. 11 Predictive performance across hyperparameter combinations (ntree, mtry, nodesize). 22
Supplementary Fig. 12 Prediction of weighted effect values of nitrogen (NUE) and phosphorus use efficiency (PUE) of the four major crops by random forest models..23

Supplementary Fig. 13 Standard deviations of predicted nitrogen (NUE, a) and phosphorus use efficiency (PUE, b) for four major crops. 24
Supplementary Fig. 14 The five major climate zones globally of sample points based on the Köppen classification[11]. 25
Supplementary Fig. 15 Global prediction of nitrogen (NUE): phosphorus (PUE) use efficiency ratios of the four major crops worldwide (a-b). The Differences in NUE: PUE ratios across different climate zones, countries, and regions (c). 26
Supplementary Fig. 16 Ratio of global major crop yields to nitrogen (N) and phosphorus (P) fertilizers loss..27
References..28

Supplementary Table 1 Comparison framework for nitrogen use efficiency (NUE) and phosphorus use efficiency (PUE) calculation approaches.

**Approaches**	**N and P difference (N(P)UE$_{diff}$)**	**^{15}N tracer (NUE$_{15N}$)**	**N and P balance (N(P)UE$_{bala}$)**
Definition	A direct, cost-effective gauge of N uptake per fertilizer unit under varied conditions	The most precise measure of N pathway through the soil-crop system, albeit at higher costs	N and P uptake divided by all N and P inputs, including biological nitrogen fixation, atmospheric deposition, fertilizer, manure and so on
Formulas	$\frac{U_{N(P)} - U_{N_0(P_0)}}{Input\ N(P)} \times 100\%$	$\frac{U_N \times \%Ndff}{Input\ N} \times 100\%$	$\frac{U_{N(P)}}{Input\ N(P) + non\ Input\ N(P)} \times 100\%$
Major focus of the assessment	Crop uptake of inorganic fertilizer N and P by using comparable no-fertilizer controls	The N uptake derived from in-season-applied N fertilizer	The use efficiency of all N and P inputs, and the fraction of N and P inputs subject to loss
Soil legacy effect	Largely excluded for short-term experiments	Not considered for single-season experiments	Partly or all included based on soil N and P status
Requirement for soil N and P status	No requirement	No requirement	Change in soil N and P stock is low or negligible compared to total N and P input and total N and P output (quasi-steady-state)
Application on spatial scales	Plot, field	Confined microplot, plot, field	Field, watershed, region, nation, or globe
Application on temporal scales	Single to multiple growing seasons	Often conducted for a single growing season	From a single growing season to multiple decades
Data source	Mostly field trials	Mostly field trials	Mostly statistical data or survey data
Appropriate use	Characterize the immediate response of the crop to N and P fertilization under different measures or practices	Partition various fates of fertilizer N at relatively small space-time scales	Evaluate the resource and environmental performances of N and P input in cropland

Note: Definition, calculation, experimental settings in cereal cropping systems (rice, wheat, maize and soybean) are shown for three methods: N(P)UE$_{diff}$, NUE$_{15N}$, N(P)UE$_{bala}$. $U_{N(P)}$ is the nitrogen (N) and phosphorus (P) uptake by mature crops from both soil and fertilizers, $U_{N_0(P_0)}$ indicates the uptake under conditions of no fertilization, $Input\ N(P)$ is the fertilizer application rate, $non\ Input\ N(P)$ represents non-fertilizer inputs, $\%N(P)dff$ denotes the fraction of N and P uptake derived from in-season-applied N and P fertilizer

Supplementary Table 2 The global mean data of crop nitrogen (N) and phosphorus (P) content[1,2], crop residue-grain ratio[3] of crops of rice, wheat, maize, soybean and overall crop.

Crop types	Rice	Wheat	Maize	Soybean	All
Grain N content (%)	1.49	1.94	1.13	3.47	1.28
Grain P content (%)	0.19	0.44	0.28	0.56	0.25
Crop residue N content (%)	0.77	0.63	0.75	1.27	0.74
Crop residue P content (%)	0.13	0.1	0.14	0.27	0.13
Crop residue–grain ratio	1.5	1.5	1	1	1

Supplementary Table 3 List of data used for calculating nitrogen from soil and phosphorus from soil.

Predictor	Unit	Original resolution	Year	Source
MAT	℃	0.5°×0.5°	1961-2018	(CRU TS)[4]
MAP	mm	0.5°×0.5°	1961-2018	(CRU TS)[4]
BD	g cm^{-3}	1 km	2014	(Shangguan et al., 2014)[5]
Sand	%	1 km	2014	(Shangguan et al., 2014)[5]
Slit	%	1 km	2014	(Shangguan et al., 2014)[5]
Clay	%	1 km	2014	(Shangguan et al., 2014)[5]
TC	g kg^{-1}	1 km	2014	(Shangguan et al., 2014)[5]
TN	g kg^{-1}	1 km	2014	(Shangguan et al., 2014)[5]
TP	g kg^{-1}	1 km	2014	(Shangguan et al., 2014)[5]
AP	mg g^{-1}	1 km	2014	(Shangguan et al., 2014)[5]
pH	/	1 km	2014	(Shangguan et al., 2014)[5]
Input N	kg ha^{-1}	/	1961-2018	FAOSTAT
Input P	kg ha^{-1}	/	1961-2018	FAOSTAT

Note: MAT, mean annual temperature; MAP, mean annual precipitation; Sand, sand content; Silt, silt content; Clay, clay content; BD, bulk density; TC, total carbon; TN, total nitrogen; TP, total phosphorus; AP, available phosphorus; pH, soil pH; Input N, nitrogen fertilizer input; Input P, phosphate fertilizer input.

Supplementary Table 4 List of global maps used for extracting missing values and scaling up.

Predictor	Unit	Original resolution	Year	Source
MAT	°C	0.5°×0.5°	2000-2022	(CRU TS)[4]
MAP	mm	0.5°×0.5°	2000-2022	(CRU TS)[4]
PET	mm	0.5°×0.5°	2000-2022	(CRU TS)[4]
ET	mm	0.25°	2000-2022	(GLEAM v3)[6]
BD	g cm^{-3}	1 km	2014	(Shangguan et al., 2014)[5]
Sand	%	1 km	2014	(Shangguan et al., 2014)[5]
Slit	%	1 km	2014	(Shangguan et al., 2014)[5]
Clay	%	1 km	2014	(Shangguan et al., 2014)[5]
TC	g kg^{-1}	1 km	2014	(Shangguan et al., 2014)[5]
TN	g kg^{-1}	1 km	2014	(Shangguan et al., 2014)[5]
TP	g kg^{-1}	1 km	2014	(Shangguan et al., 2014)[5]
AP	mg g^{-1}	1 km	2014	(Shangguan et al., 2014)[5]
pH	/	1 km	2014	(Shangguan et al., 2014)[5]
IND	Kg km^{-2} yr^{-1}	2°×2.5°	2004-2016	(Ackerman et al., 2019)[7]
OND	Kg km^{-2} yr^{-1}	2°×2.5°	2004-2016	(Ackerman et al., 2019)[7]
Tillage	/	5 arcmin	2000-2020	(Porwollik et al., 2019)[8]
Crop type	/	10 km	2018	SPAM 2010 V2r0[9]
Grain yield	kg ha^{-1}	/	2018	FAOSTAT (2018)
Fertilizer	kg ha^{-1}	/	2018	IFA (2018)
GDPPC	US$	/	2000-2022	World bank data
PD	People km^{-2}	/	2000-2022	World bank data
UR	%	/	2000-2022	World bank data
MYS	year	/	2000-2022	Human development data

Note: The fertilizer input intensities data provided by the IFA dataset were last recorded in 2019, with notably limited data for that year[10]. Therefore, 2018 fertilizer input data were chosen for prediction. MAT, mean annual temperature; MAP, mean annual precipitation; Sand, sand content; Silt, silt content; Clay, clay content; BD, bulk density; TC, total carbon; TN, total nitrogen; TP, total phosphorus; AP, available phosphorus; Input N, nitrogen fertilizer input; Input P, phosphate fertilizer input.

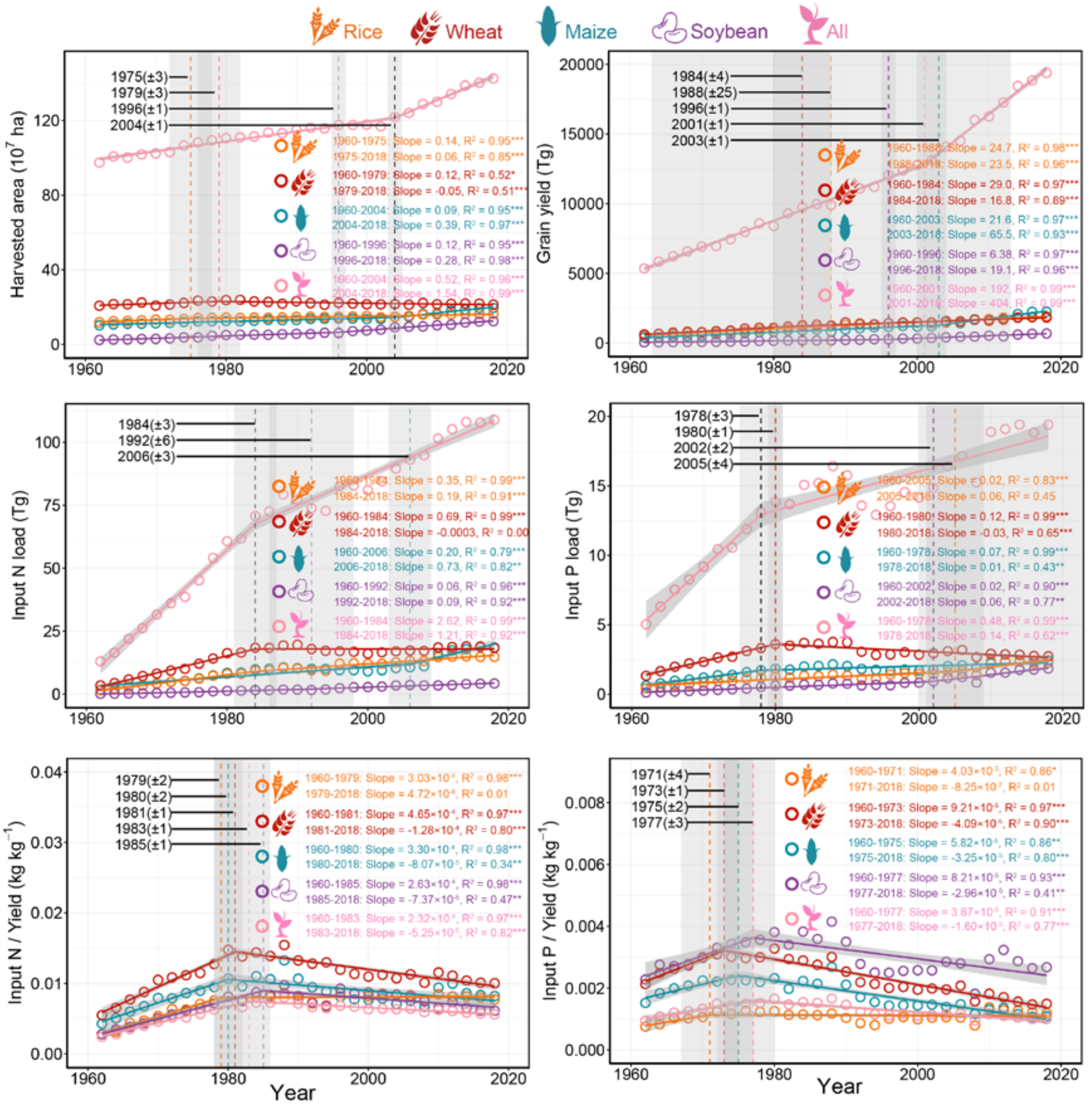

Supplementary Fig. 1 Trends in global harvested area, grain yield, input N(P) loads and input N(P) / Yield over time.

Trends in harvested area, grain yield, input N(P) loads and input N(P) / Yield over time were fitted using a segmented fit, with solid lines indicating segmented fitting lines and corresponding shaded areas representing 95% confidence interval of the fitting lines. The significance of the non-zero coefficients in the segmented model fitting is evaluated through a two-tailed t-test. If $p > 0.05$, it indicates that there is no obvious trend of value over time. The data are marked in the upper left corner are the values of vertical dashed lines and shaded areas, indicating breakpoints (turning points) and standard error, respectively. The color of dashed lines is consistent with that of the crop legend, and black indicates that the breakpoints of multiple crops are consistent. *, $p < 0.05$; **, $p < 0.01$; ***, $p < 0.001$. Data were obtained from the Food and Agriculture Organization of the United Nations and the International Fertilizer Association.

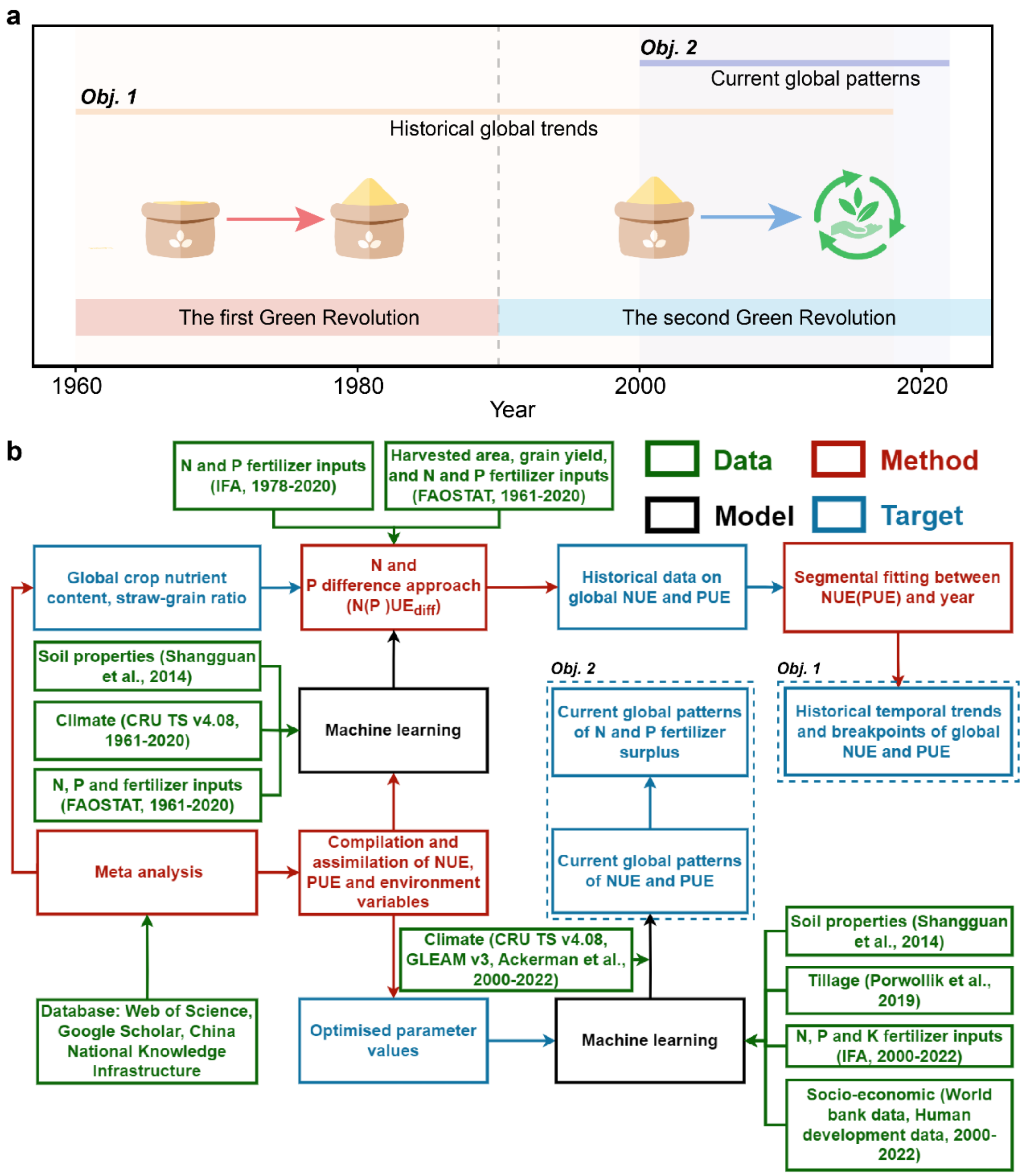

Supplementary Fig. 2 Research period (a) and workflow (b) on global inorganic fertilizer nitrogen use efficiency (NUE) and phosphorus use efficiency (PUE) trends and patterns.

Our research involved the two periods of the first Green Revolution and the second Green Revolution. During these periods, we first complied and assimilated the NUE, PUE and environment variables at each sampling point, sourced from meta-analysis, to acquire optimized parameter values and the predicted model of nitrogen(phosphorus) from soil. N and P difference approach were employed to compute historical data on global NUE and PUE from 1960 to 2018, utilizing the predicted model of nitrogen(phosphorus) from soil, parameters from IFA and FAOSTAT. These historical data were utilized to depict the historical temporal trends and respective breakpoints of global NUE and PUE through segmental fitting. Moreover, random forest model predicted the patterns of global NUE and PUE from 2000 to 2022, using the optimized parameters, climate (n = 6), soil (n = 9), agriculture management (n = 7) and socio-economic (n = 4) factors. The predicted

values were further applied to calculate the global N and P surplus.

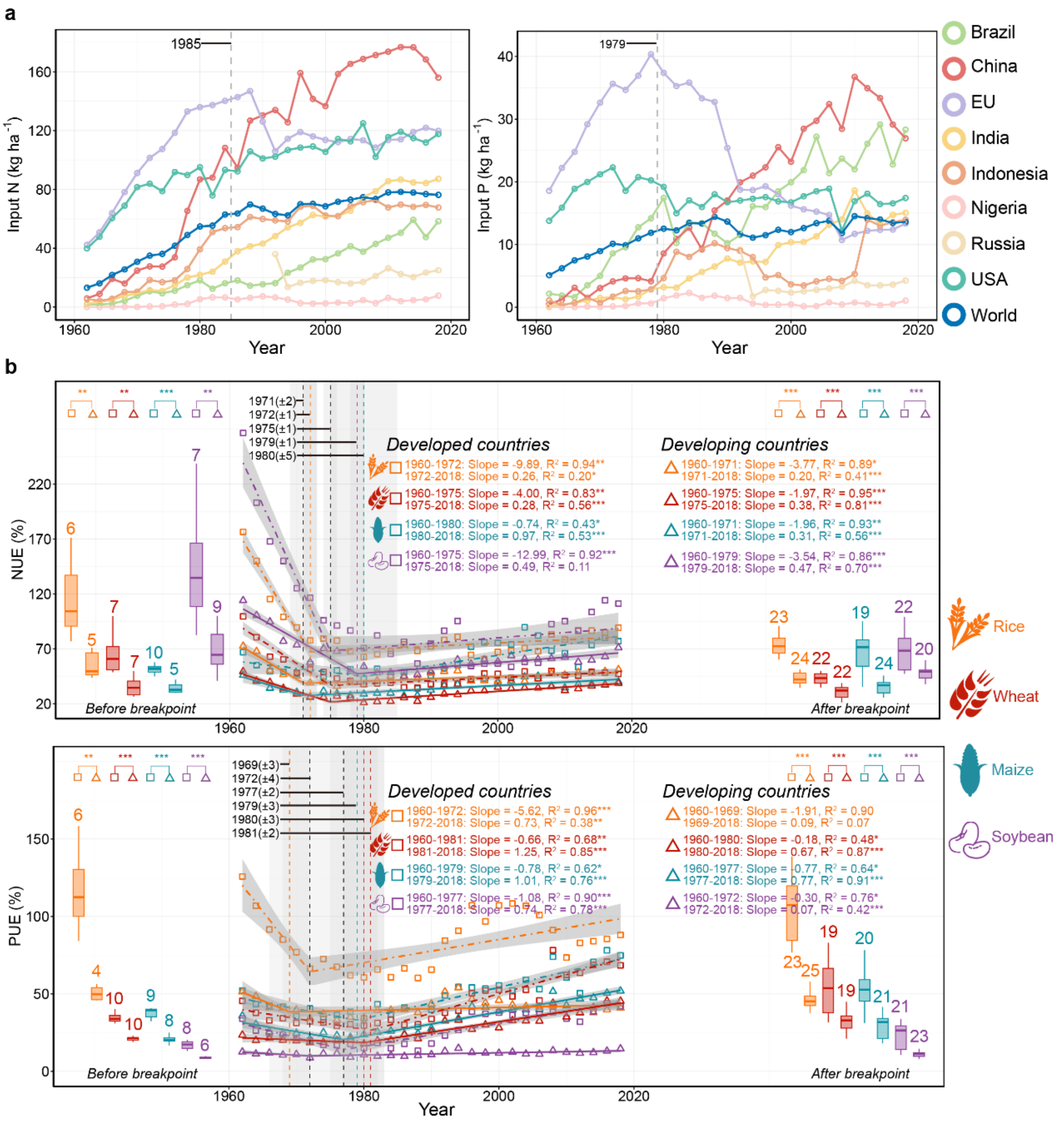

Supplementary Fig. 3 Nitrogen (N), phosphorus (P) fertilizer input intensities (a), and nitrogen (NUE), phosphorus use efficiency (PUE) (b) for major countries on temporal scales.

The gray dashed line indicates the breakpoint of the temporal scales, referenced in Fig. 2. Ranking of major countries is based on crop cultivation area, and European Union (EU) participation is based on January 2024 including 27 countries and excluding the United Kingdom (UK), which has left the EU in 2020. Trends in utilization efficiencies (**b**) over time were fitted using a segmented fit, with vertical dashed lines indicating breakpoints (turning points) and shaded areas representing 95% confidence intervals. The fit trend lines for developed and developing countries are represented by dotted lines and solid lines, respectively. The significance of the non-zero coefficients in the segmented model fitting is evaluated through a two-tailed t-test. If $p > 0.05$, it indicates that there is no obvious trend of value over time. The data are marked in the upper left corner are the values of vertical dashed lines and shaded areas, indicating breakpoints (turning points) and standard error, respectively. The color of dashed lines is consistent with that of the crop legend, and black indicates that the breakpoints of multiple crops are consistent. The significance test of difference of major

crops in developed and developing countries is represented by box plot based on wilcox method, using two-tailed test. For the boxplot, the straight line in the centre represents the median, or second quartile (Q2), the top edge of the box represents the third quartile (Q3) and the bottom edge of the box represents the first quartile (Q1). Top or bottom number on boxplot represents the number of data points in each box. *, $p < 0.05$; **, $p < 0.01$; ***, $p < 0.001$.

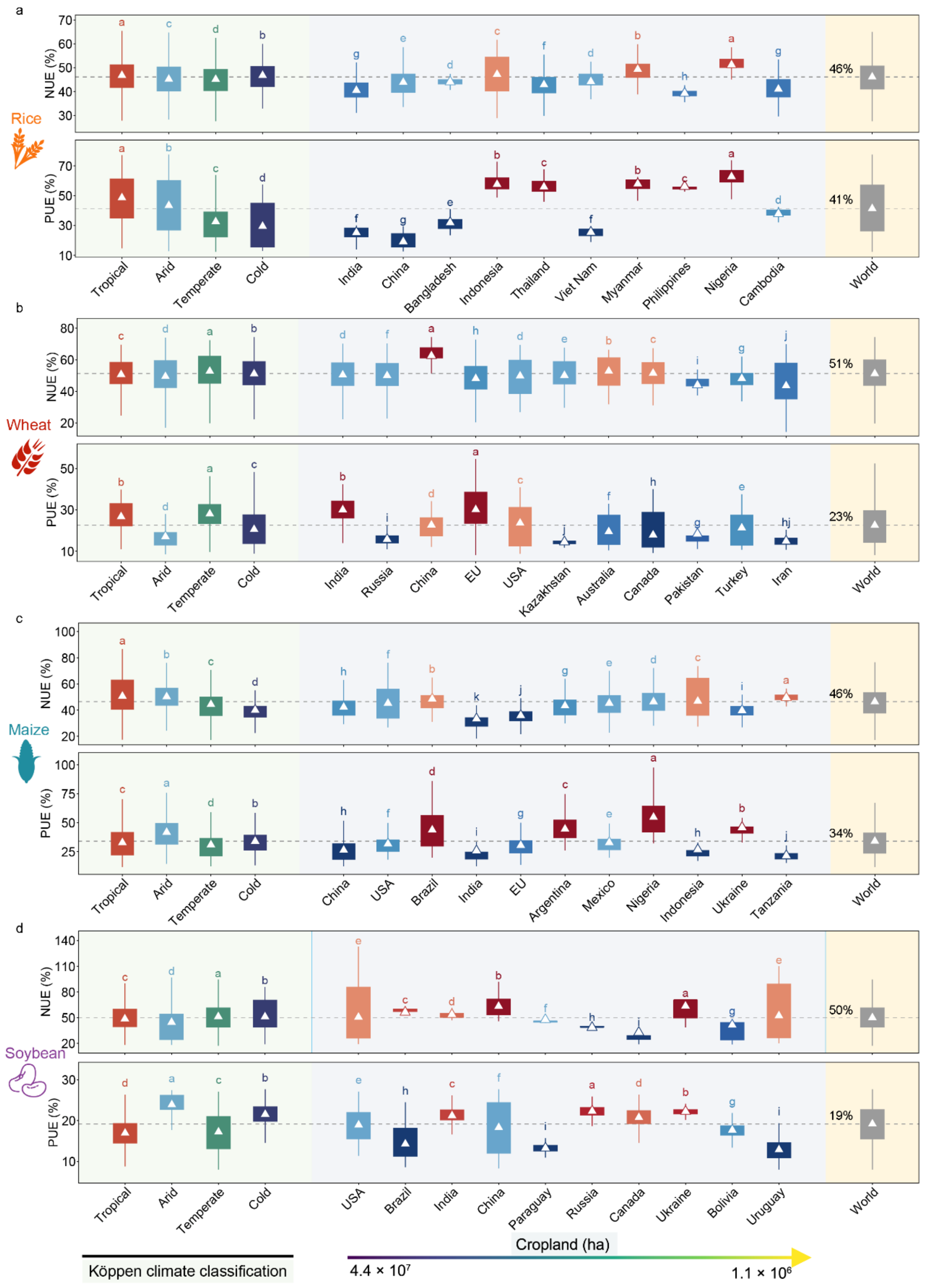

Supplementary Fig. 4 Nitrogen (NUE) and phosphorus (PUE) use efficiencies for rice (a), wheat (b), maize (c), soybean (d) in different climatic zones, major countries or regions, and globally.

The box plots display the mean (represented by a triangle) along with the 25th (Q1) and 75th (Q3) percentiles (box boundaries), and the whiskers indicate the Q1 − 1.5 IQR and Q3 + 1.5 IQR of the observations. We used Dunn's Kruskal-Wallis multiple comparison to obtain the difference of

multiple groups of box plots, and the significant p-values of the data adjusted with the Bonferroni method. Different letters on the left represent significant differences $p < 0.05$. Each box summarizes statistical distributions derived from a global predictive raster dataset (n = 748604) across different countries and regions. Climate zone delineation basis based on Köppen climate classification, visualized in Supplementary Fig. 14. Different letters represent significant differences at the level $p < 0.05$.

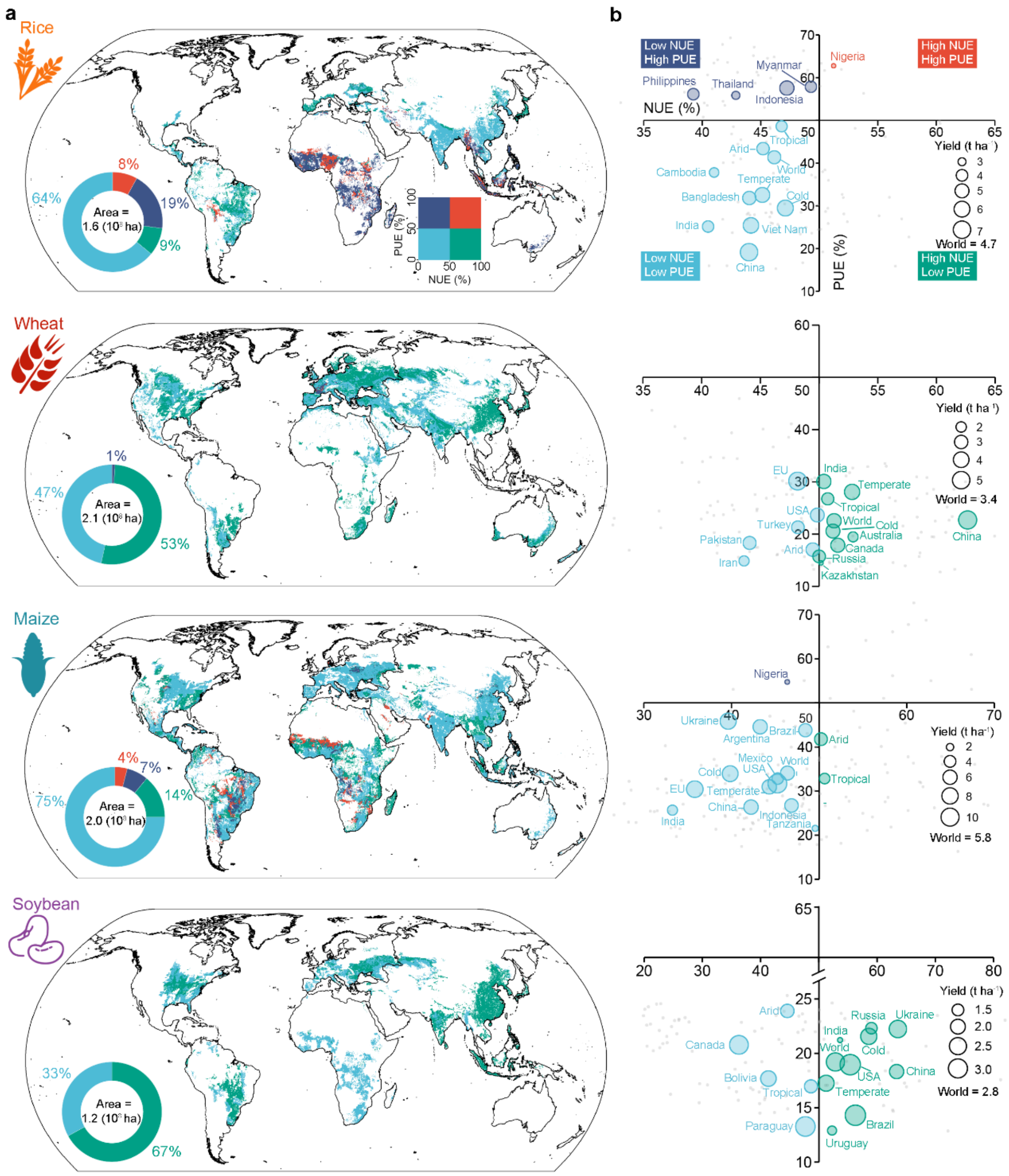

Supplementary Fig. 5 Global-scale synergistic effects of nitrogen (NUE) and phosphorus use efficiency (PUE) of four major crops: rice, wheat, maize and soybean.

The four-quadrant division of NUE and PUE is based on a 50% threshold **(a)**, implying the destination of most of the nitrogen and phosphorus fertilizers **(b)**. Climate zone delineation basis based on Köppen climate classification, visualized in Supplementary Fig. 14.

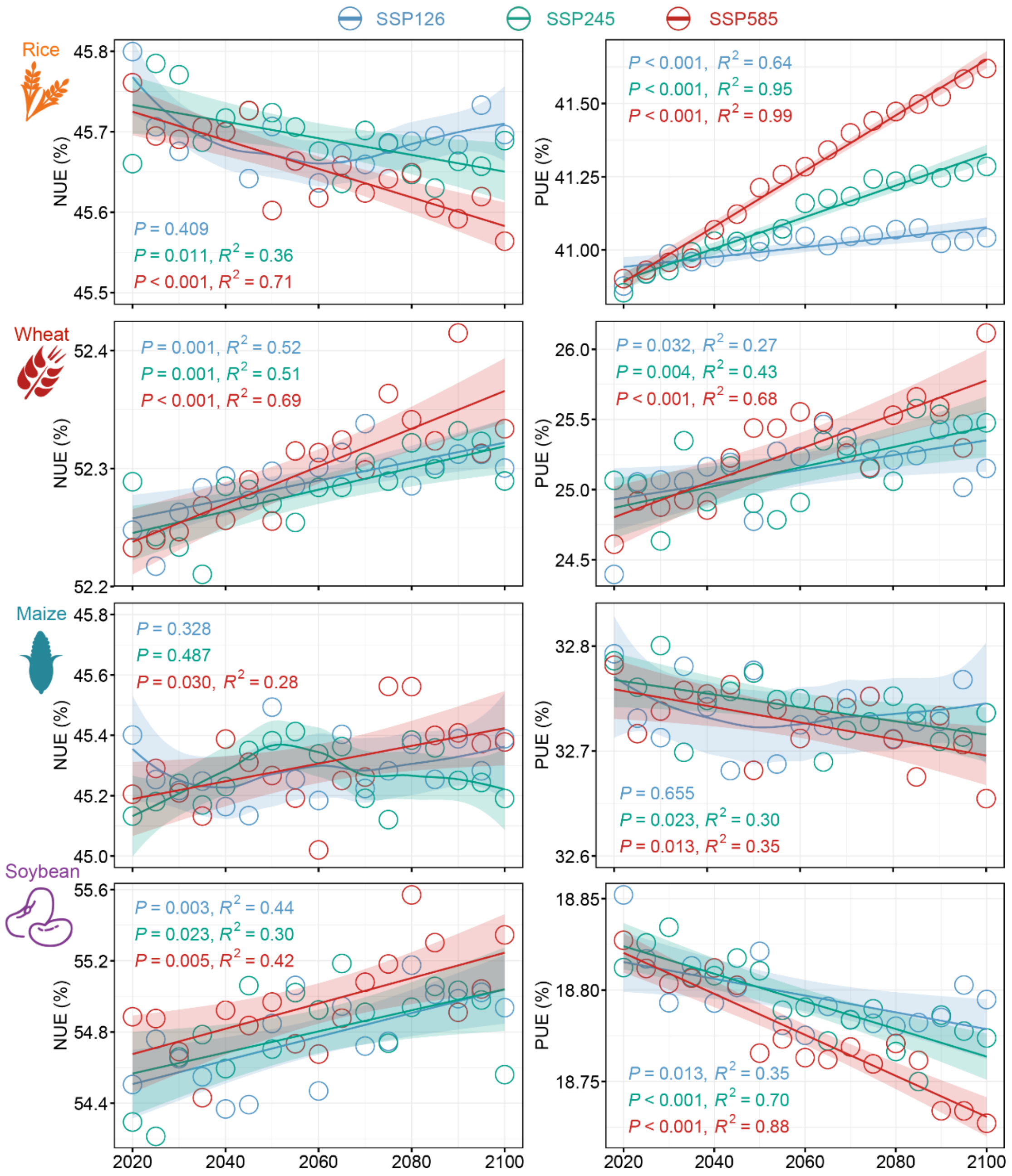

Supplementary Fig. 6 Temporal trends of nitrogen (NUE) and phosphorus use efficiency (PUE) of four major crops (rice, wheat, maize, soybean) under SSP126 (sustainable development-low forcing), SSP245 (moderate development-medium forcing), and SSP585 (fossil-fueled development-high forcing) scenarios.

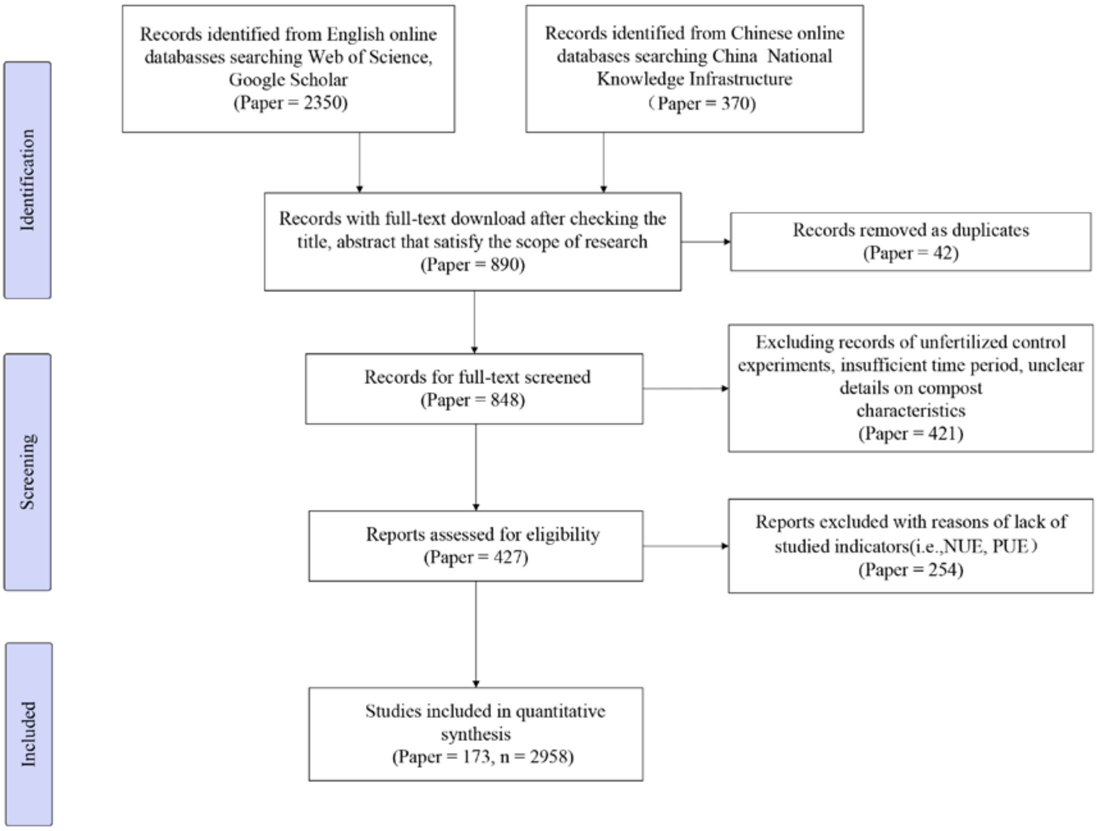

Supplementary Fig. 7 The Preferred Reporting Items for Systematic Reviews and Meta-Analyses (PRISMA) for the meta-analysis

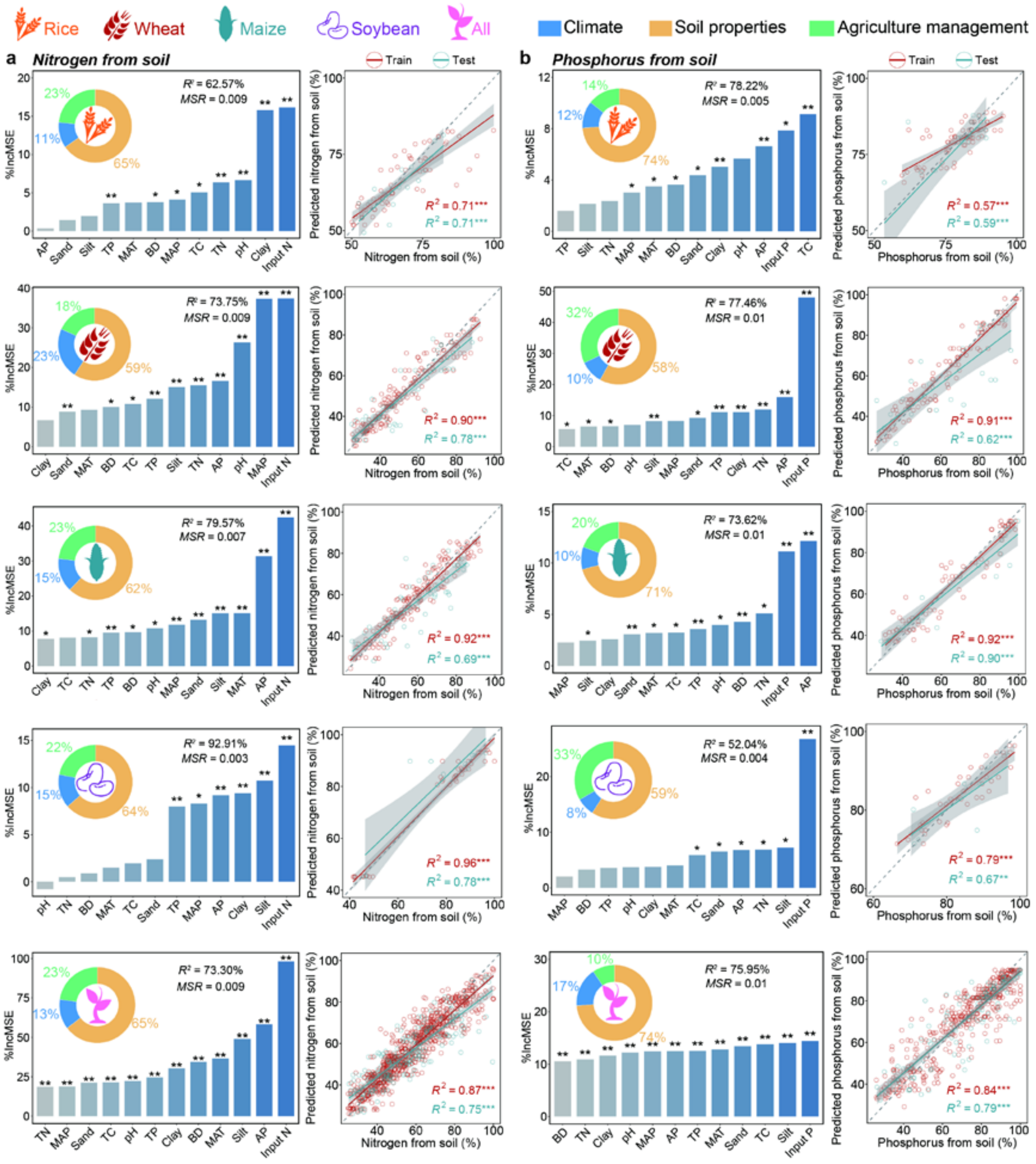

Supplementary Fig. 8 Prediction of weighted effect values of nitrogen (a) and phosphorus (b) from soil of the four major crops (rice, wheat, maize, soybean) and all crops by random forest models.

MAT, mean annual temperature; MAP, mean annual precipitation; Sand, sand content; Silt, silt content; Clay, clay content; BD, bulk density; TC, total carbon; TN, total nitrogen; TP, total phosphorus; AP, available phosphorus; Input N, nitrogen fertilizer input; Input P, phosphate fertilizer input. The significance of the variables in Figure is measured by the "percentage of increase of mean square error" (%IncMSE) value in Random Forest, where higher %IncMSE values imply more important variables and identify the significance of each variable. *: $p < 0.05$; **: $p < 0.01$; ***: $p < 0.001$; ****: $p < 0.0001$. The values at the top of the graph are the Var explained (R^2) and the Mean of squared residuals (*MSR*) for the full model. We counted the proportions of each class of factors (Fig. 3) in the random forest and represented them in a circle plot. We demonstrate the prediction performance of 80 percent of the data for the training set versus 20 percent of the test set, where R^2 represents the correlation between the observed nitrogen(phosphorus) from soil and the

predicted nitrogen(phosphorus) from soil.

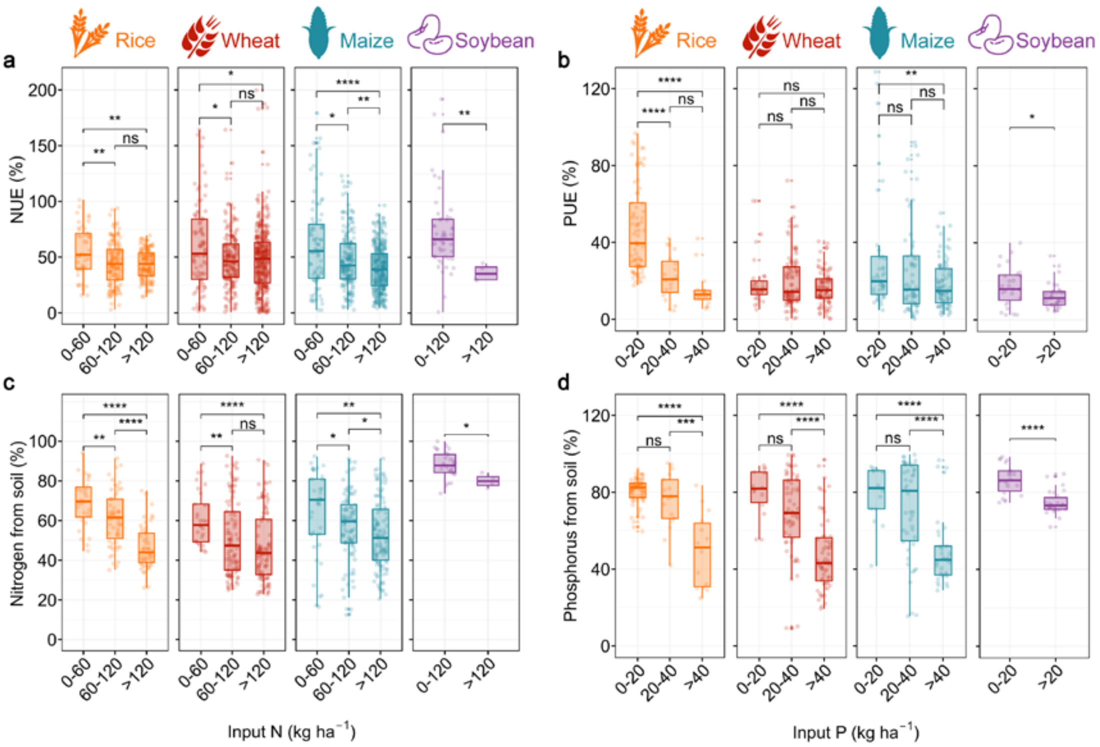

Supplementary Fig. 9 Nitrogen (NUE) and phosphorus use efficiency (PUE) and the proportion of nitrogen (nitrogen from soil) and phosphorus (phosphorus from soil) uptake from the soil for four major crops at different fertilizer input intensities.

A two-sided Wilcoxon signed-rank test was employed to analyze differences in NUE (**a**) and soil-derived nitrogen (**c**) among crop species (n = 356, 669, 625, and 62 for rice, wheat, maize, and soybean, respectively); for PUE (**b**) and soil-derived phosphorus (**d**), the sample sizes were n = 112, 223, 206, and 63, respectively. ****, $p < 0.0001$; ***, $p < 0.001$; **, $p < 0.01$; *, $p < 0.05$.

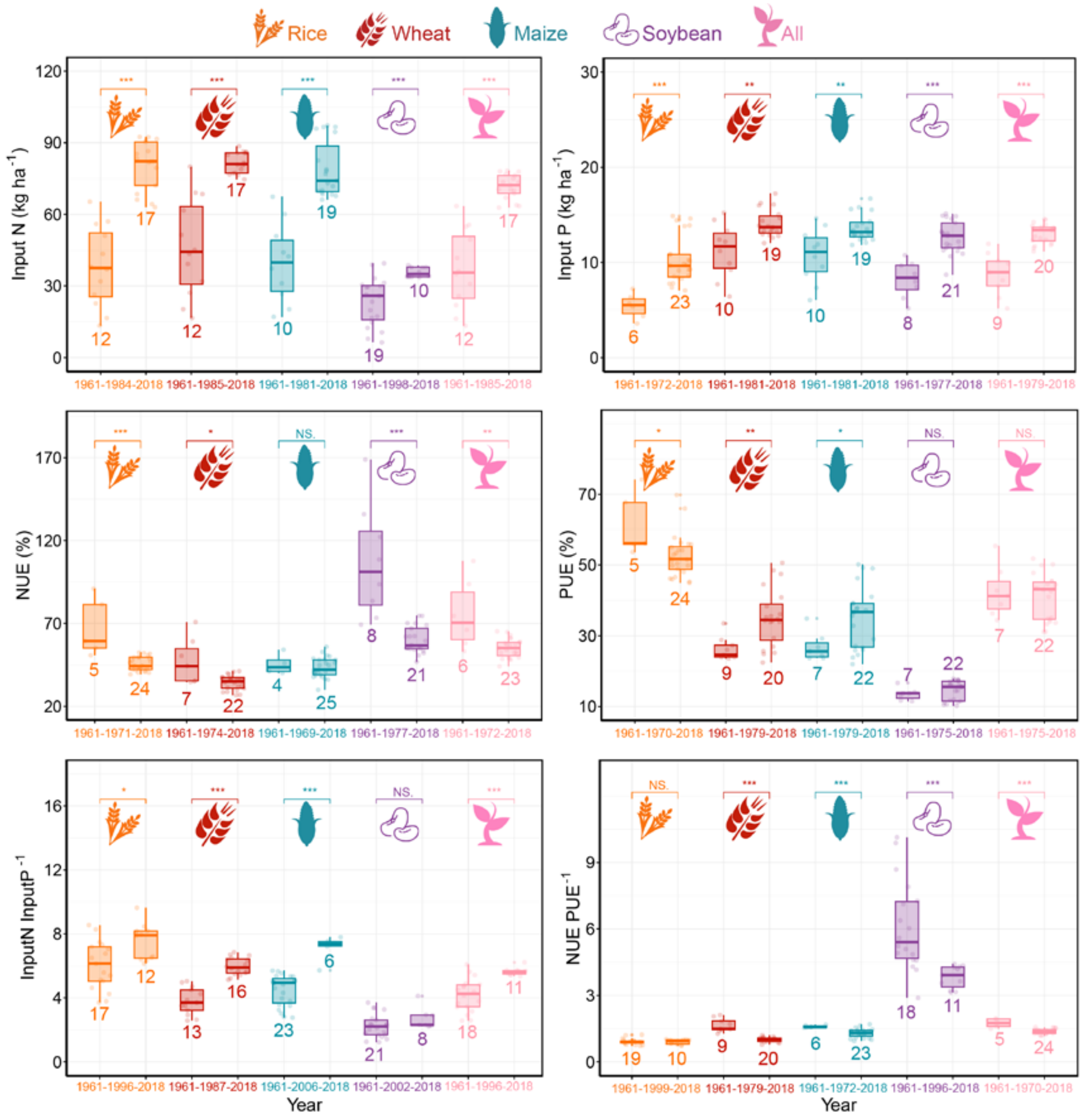

Supplementary Fig. 10 The significance test of difference in rice, wheat, maize, soybean and all crops across years before and after their respective breakpoints. It was determined through segmented fitting (Fig. 2). The significance test of difference of major crops in developed and developing countries is represented by box plot based on wilcox method, using two-tailed test. For the boxplot, the straight line in the centre represents the median, or second quartile (Q2), the top edge of the box represents the third quartile (Q3) and the bottom edge of the box represents the first quartile (Q1). Top or bottom number on boxplot represents the number of data points in each box. *, $p < 0.05$; **, $p < 0.01$; ***, $p < 0.001$.

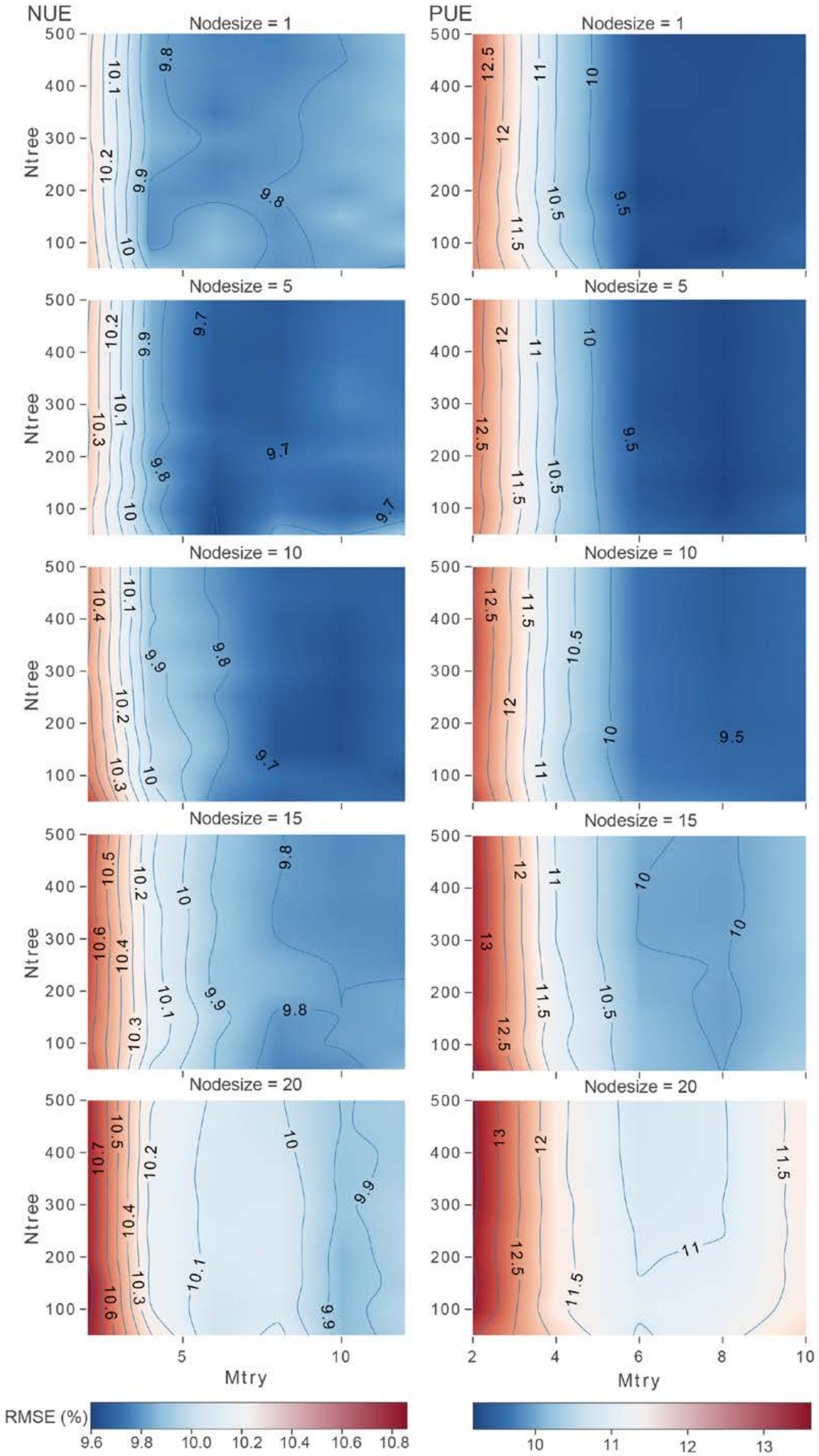
NUE
PUE
Nodesize = 1
Nodesize = 5
Nodesize = 10
Nodesize = 15
Nodesize = 20
Ntree
Mtry
RMSE (%)

Supplementary Fig. 11 Predictive performance across hyperparameter combinations (ntree, mtry, nodesize).

In Random Forest model, the ntree parameter defines the total number of decision trees, balancing model stability and computational cost; mtry determines the number of randomly selected features evaluated at each node split, influencing feature diversity and overfitting risk; while nodesize sets the minimum observations required in terminal leaves, controlling tree depth and granularity to prevent over- or underfitting.

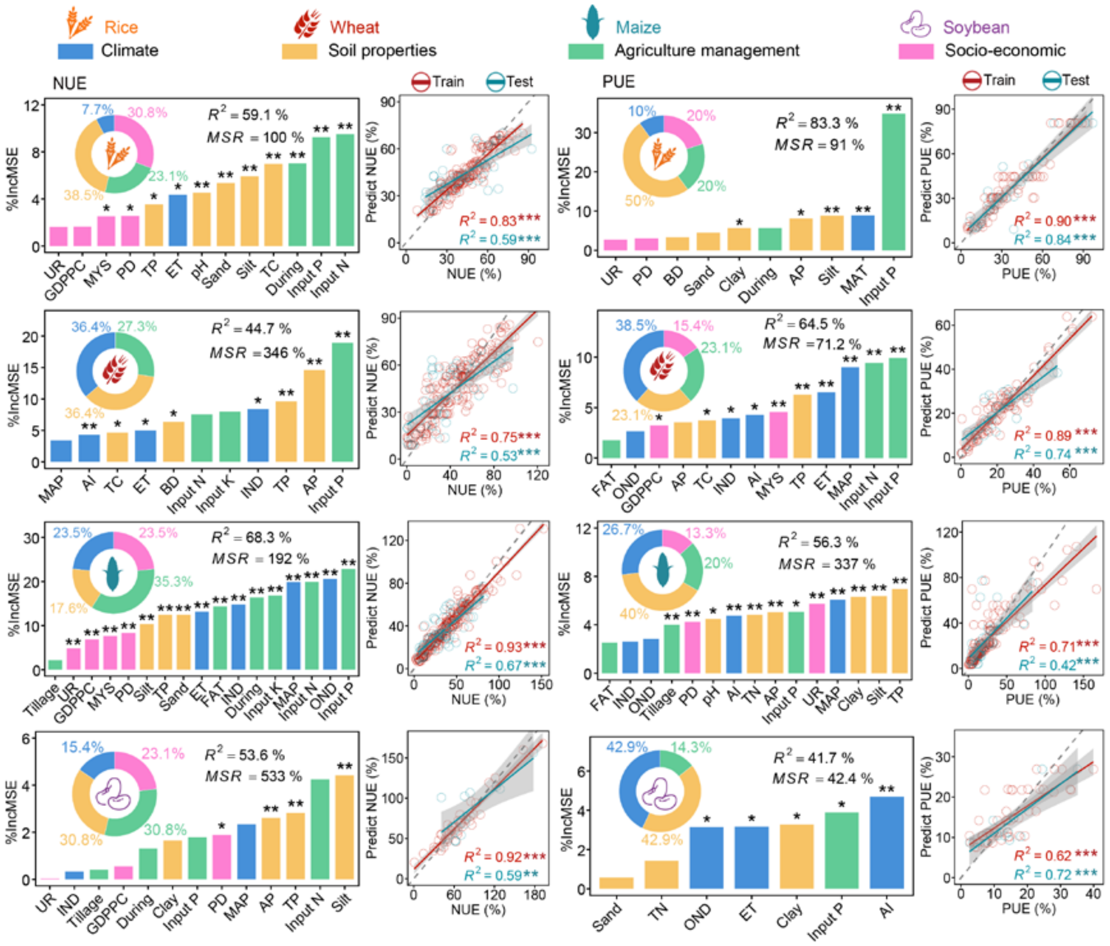

Supplementary Fig. 12 Prediction of weighted effect values of nitrogen (NUE) and phosphorus use efficiency (PUE) of the four major crops by random forest models. MAT, mean annual temperature; MAP, mean annual precipitation; ET, evapotranspiration; AI, aridity index; IND, inorganic nitrogen deposition; OND, organic nitrogen deposition; Sand, sand content; Silt, silt content; Clay, clay content; BD, bulk density; TC, total carbon; TN, total nitrogen; TP, total phosphorus; AP, available phosphorus; Tillage, no-till or not; During, planting years; Input N, nitrogen fertilizer input; Input P, phosphate fertilizer input; Input K, potash fertilizer input; FAT, Fertilizer application types; GDPPC, gross domestic product per capita; PD, population density; UR, urbanization rate; MYS, mean years of schooling. The significance of the variables in Figure is measured by the "percentage of increase of mean square error" (%IncMSE) value in Random Forest, where higher %IncMSE values imply more important variables and identify the significance of each variable. *: $p < 0.05$; **: $p < 0.01$; ***: $p < 0.001$; ****: $p < 0.0001$. The values at the top of the graph are the Var explained (R^2) and the Mean of squared residuals (*MSR*) for the full model. We counted the proportions of each class of factors (Fig. 3) in the random forest and represented them in a circle plot. We demonstrate the prediction performance of 80 percent of the data for the training set versus 20 percent of the test set, where R^2 represents the correlation between the Observed NUE / PUE and the predicted NUE / PUE.

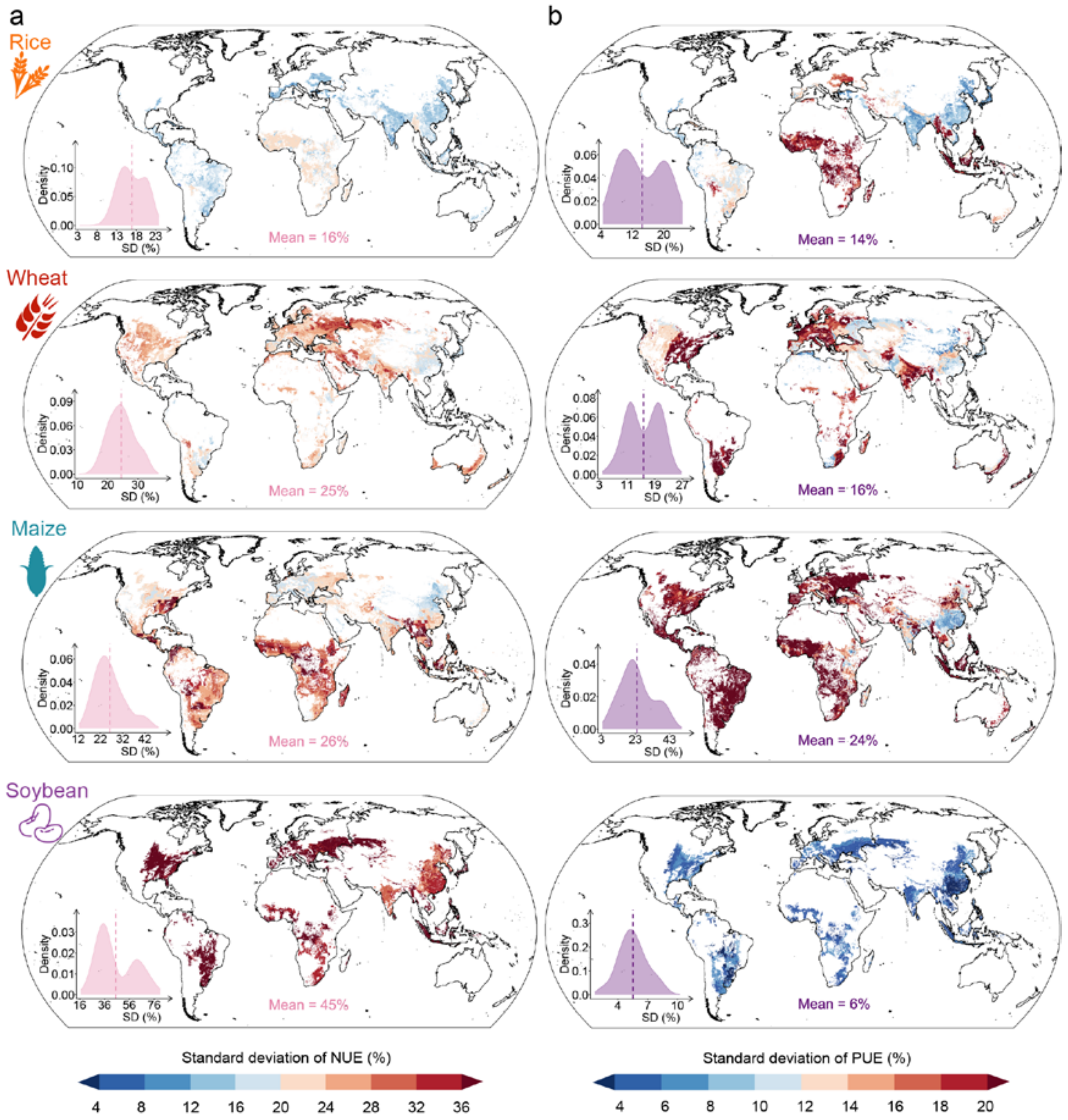

Supplementary Fig. 13 Standard deviations of predicted nitrogen (NUE, a) and phosphorus use efficiency (PUE, b) for four major crops.

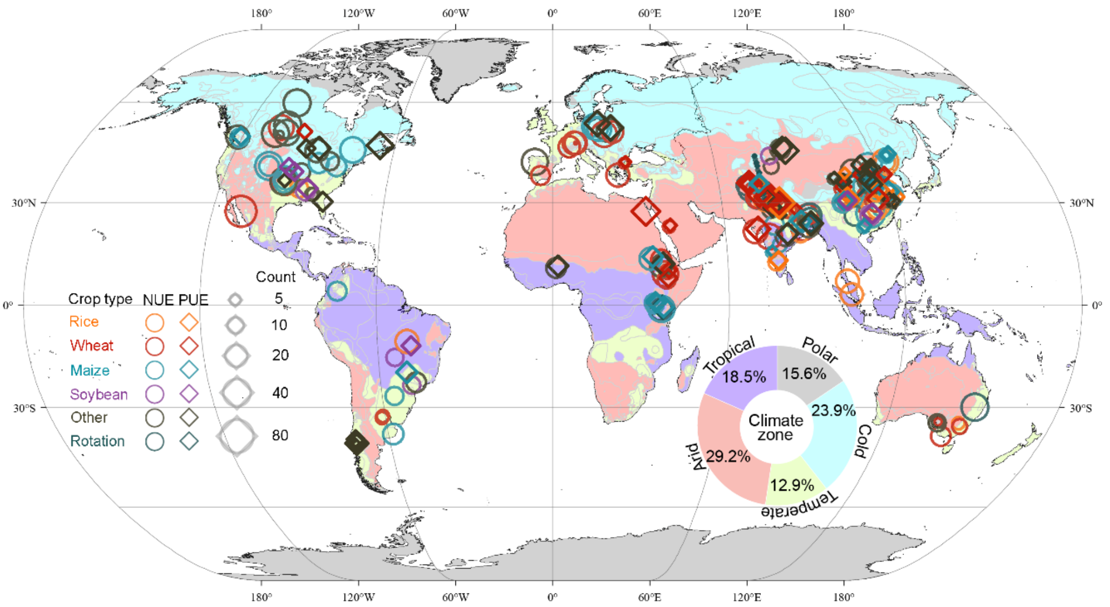

Supplementary Fig. 14 The five major climate zones globally of sample points based on the Köppen classification[11].

Count represents the frequency of the collected samples under the same location. NUE, nitrogen use efficiency; PUE, phosphorus use efficiency.

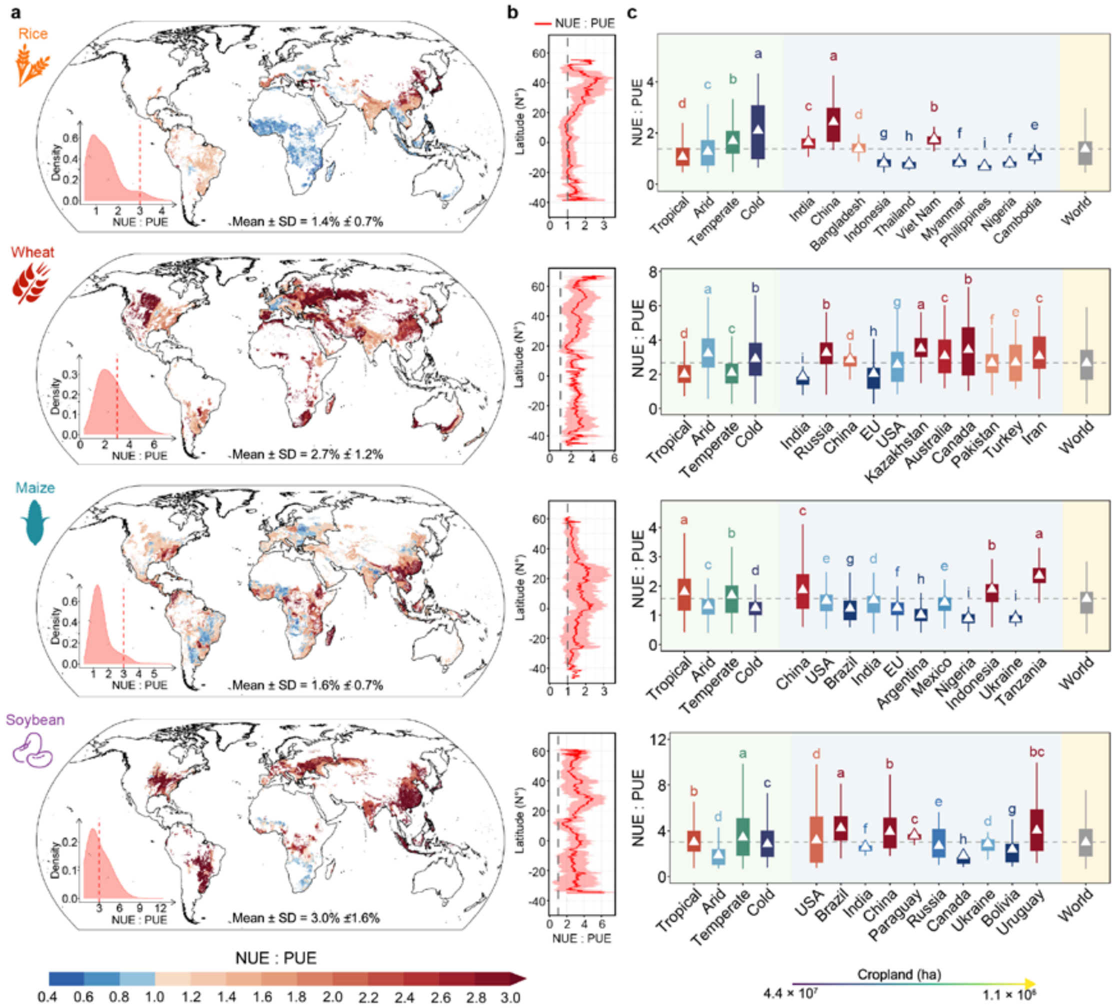

Supplementary Fig. 15 Global prediction of nitrogen (NUE): phosphorus (PUE) use efficiency ratios of the four major crops worldwide (a-b). The Differences in NUE: PUE ratios across different climate zones, countries, and regions (c).

The solid lines and shaded areas in the latitudinal pattern map represent the latitudinal mean and the latitudinal standard deviation respectively. The box plots display the mean (represented by a triangle) along with the 25th (Q1) and 75th (Q3) percentiles (box boundaries), and the whiskers indicate the Q1 − 1.5 IQR and Q3 + 1.5 IQR of the observations. We used Dunn's Kruskal-Wallis multiple comparison to obtain the difference of multiple groups of box plots, and the significant p-values of the data adjusted with the Bonferroni method. Different letters on the left represent significant differences $p < 0.05$. Each box summarizes statistical distributions derived from a global predictive raster dataset (n = 748604) across different countries and regions. Climate zone delineation basis based on Köppen climate classification, visualized in Supplementary Fig. 14. Ranking of major countries is based on crop cultivation area, and European Union (EU) participation is based on January 2024 including 27 countries and excluding the United Kingdom (UK), which has left the EU in 2020. Different letters represent significant differences at the level $p < 0.05$.

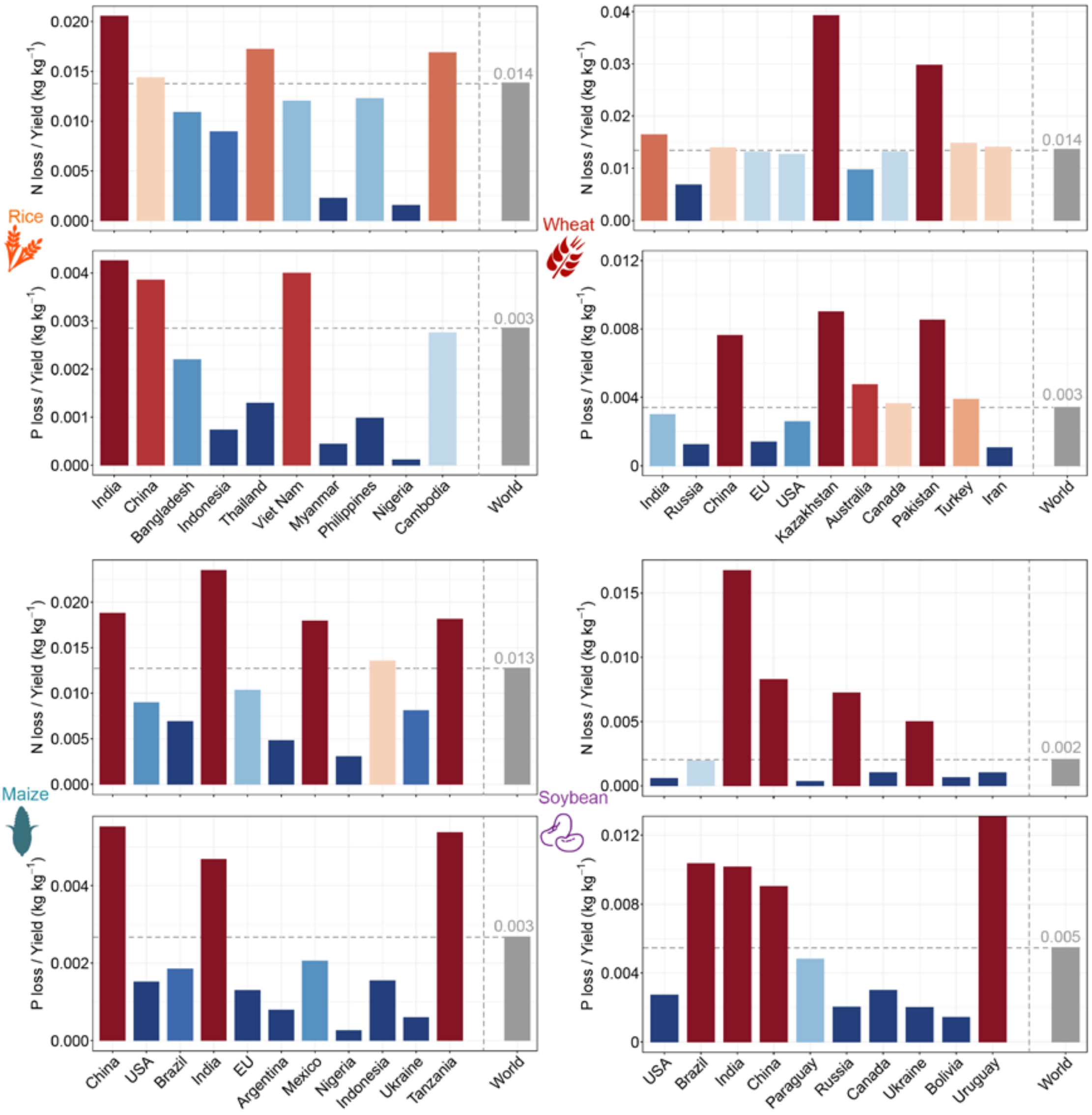

Supplementary Fig. 16 Ratio of global major crop yields to nitrogen (N) and phosphorus (P) fertilizers loss.

Each bar summarizes statistical distributions derived from a global predictive raster dataset (n = 748604) across different countries and regions. Ranking of major countries is based on crop cultivation area, and European Union (EU) participation is based on January 2024 including 27 countries and excluding the United Kingdom (UK), which has left the EU in 2020.

References

1. Zhang, X. *et al.* Quantification of global and national nitrogen budgets for crop production. *Nat. Food* **2**, 529-540 (2021).
2. Zou, T., Zhang, X. & Davidson, E. Global trends of cropland phosphorus use and sustainability challenges. *Nature* **611**, 81-87 (2022).
3. Lal, R. World crop residues production and implications of its use as a biofuel. *Environ. Int.* **31**, 575-584 (2005).
4. Harris, I., Osborn, T. J., Jones, P. & Lister, D. Version 4 of the CRU TS monthly high-resolution gridded multivariate climate dataset. *Sci. Data* **7**, 109 (2020).
5. Shangguan, W., Dai, Y., Duan, Q., Liu, B. & Yuan, H. A global soil data set for earth system modeling. *J. Adv. Model Earth Sy.* **6**, 249-263 (2014).
6. Miralles, D. G. *et al.* Global land-surface evaporation estimated from satellite-based observations. *Hydrol. Earth Syst. Sci.* **15**, 453-469 (2011).
7. Ackerman, D., Millet, D. B. & Chen, X. Global estimates of inorganic nitrogen deposition across four decades. *Global Biogeochem Cy.* **33**, 100-107 (2019).
8. Porwollik, V., Rolinski, S., Heinke, J. & Müller, C. Generating a rule-based global gridded tillage dataset. *Earth Syst. Sci. Data* **11**, 823-843 (2019).
9. Institute, I. F. P. R. (Harvard Library Cambridge, MA, 2019).
10. Ludemann, C., Gruere, A., Heffer, P. & Dobermann, A. Global data on fertilizer use by crop and by country. *Sci. Data* **9**, 501 (2022).
11. Peel, M. C., Finlayson, B. L. & McMahon, T. A. Updated world map of the Köppen-Geiger climate classification. *Hydrol. Earth Syst. Sci.* **11**, 1633-1644 (2007).
