# Supplementary material for: Global-scale prevalence of low nutrient use efficiency across major crops
Source: Nat Commun. 2025 Dec 10;16:11036. doi: 10.1038/s41467-025-66019-w (PMC12695879; doi:10.1038/s41467-025-66019-w)
Supplement: Supplementary file 3 — Reporting Summary [file 41467_2025_66019_MOESM3_ESM.pdf]

nature portfolio

Corresponding author(s): Linchuan Fang
Last updated by author(s): Sep 25, 2025

Reporting Summary

Nature Portfolio wishes to improve the reproducibility of the work that we publish. This form provides structure for consistency and transparency in reporting. For further information on Nature Portfolio policies, see our Editorial Policies and the Editorial Policy Checklist.

Statistics

For all statistical analyses, confirm that the following items are present in the figure legend, table legend, main text, or Methods section.

n/a	Confirmed	
☐	☒	The exact sample size (*n*) for each experimental group/condition, given as a discrete number and unit of measurement
☐	☒	A statement on whether measurements were taken from distinct samples or whether the same sample was measured repeatedly
☐	☒	The statistical test(s) used AND whether they are one- or two-sided<br>*Only common tests should be described solely by name; describe more complex techniques in the Methods section.*
☐	☒	A description of all covariates tested
☐	☒	A description of any assumptions or corrections, such as tests of normality and adjustment for multiple comparisons
☐	☒	A full description of the statistical parameters including central tendency (e.g. means) or other basic estimates (e.g. regression coefficient) AND variation (e.g. standard deviation) or associated estimates of uncertainty (e.g. confidence intervals)
☐	☒	For null hypothesis testing, the test statistic (e.g. *F*, *t*, *r*) with confidence intervals, effect sizes, degrees of freedom and *P* value noted<br>*Give P values as exact values whenever suitable.*
☒	☐	For Bayesian analysis, information on the choice of priors and Markov chain Monte Carlo settings
☒	☐	For hierarchical and complex designs, identification of the appropriate level for tests and full reporting of outcomes
☐	☒	Estimates of effect sizes (e.g. Cohen's *d*, Pearson's *r*), indicating how they were calculated

Our web collection on statistics for biologists contains articles on many of the points above.

Software and code

Policy information about availability of computer code

Data collection	To quantitatively evaluate global trends and drivers of the NUE and PUE of major crops (rice, wheat, maize, and soybean), we undertook a comprehensive data collection initiative (Supplementary Fig. 7) by a systematic literature review of peer-reviewed articles obtained from the Web of Science (https://www.web of science.com/), Google Scholar (https://scholar.google.com/), and China National Knowledge Infrastructure (https://oversea.cnki.net/index/), up to April 2023 (Supplementary Fig. 7).
Data analysis	ALL statistical analyses were performed using R 4.2.2 (R(R Development Core Team, 2020). Detail were report inin Methods section

For manuscripts utilizing custom algorithms or software that are central to the research but not yet described in published literature, software must be made available to editors and reviewers. We strongly encourage code deposition in a community repository (e.g. GitHub). See the Nature Portfolio guidelines for submitting code & software for further information.

Data

Policy information about availability of data

All manuscripts must include a data availability statement. This statement should provide the following information, where applicable:

- Accession codes, unique identifiers, or web links for publicly available datasets
- A description of any restrictions on data availability
- For clinical datasets or third party data, please ensure that the statement adheres to our policy

Provide your data availability statement here.

Research involving human participants, their data, or biological material

Policy information about studies with human participants or human data. See also policy information about sex, gender (identity/presentation), and sexual orientation and race, ethnicity and racism.

Reporting on sex and gender	*Use the terms sex (biological attribute) and gender (shaped by social and cultural circumstances) carefully in order to avoid confusing both terms. Indicate if findings apply to only one sex or gender; describe whether sex and gender were considered in study design; whether sex and/or gender was determined based on self-reporting or assigned and methods used.* *Provide in the source data disaggregated sex and gender data, where this information has been collected, and if consent has been obtained for sharing of individual-level data; provide overall numbers in this Reporting Summary. Please state if this information has not been collected.* *Report sex- and gender-based analyses where performed, justify reasons for lack of sex- and gender-based analysis.*
Reporting on race, ethnicity, or other socially relevant groupings	*Please specify the socially constructed or socially relevant categorization variable(s) used in your manuscript and explain why they were used. Please note that such variables should not be used as proxies for other socially constructed/relevant variables (for example, race or ethnicity should not be used as a proxy for socioeconomic status).* *Provide clear definitions of the relevant terms used, how they were provided (by the participants/respondents, the researchers, or third parties), and the method(s) used to classify people into the different categories (e.g. self-report, census or administrative data, social media data, etc.)* *Please provide details about how you controlled for confounding variables in your analyses.*
Population characteristics	*Describe the covariate-relevant population characteristics of the human research participants (e.g. age, genotypic information, past and current diagnosis and treatment categories). If you filled out the behavioural & social sciences study design questions and have nothing to add here, write "See above."*
Recruitment	*Describe how participants were recruited. Outline any potential self-selection bias or other biases that may be present and how these are likely to impact results.*
Ethics oversight	*Identify the organization(s) that approved the study protocol.*

Note that full information on the approval of the study protocol must also be provided in the manuscript.

Field-specific reporting

Please select the one below that is the best fit for your research. If you are not sure, read the appropriate sections before making your selection.

☐ Life sciences ☐ Behavioural & social sciences ☒ Ecological, evolutionary & environmental sciences

For a reference copy of the document with all sections, see nature.com/documents/nr-reporting-summary-flat.pdf

Ecological, evolutionary & environmental sciences study design

All studies must disclose on these points even when the disclosure is negative.

Study description	we aim to address these knowledge gaps by compiling a comprehensive global database on nutrient use efficiency (NUE and PUE), comprising 3,360 observations across 205 countries and regions (Fig. 1). We analyze trends and patterns of NUE and PUE using the N(P)UEdiff framework for major crops, utilizing dynamic national-scale data that includes crop yields, cropland areas, fertilizer N and P input intensities, nutrient uptake by crops, and residue-grain ratios. Our focus on key global crops—specifically rice, wheat, maize, and soybean—that collectively account for over half of global crop production and 49% of cropland area.
Research sample	we develop machine learning models utilizing point-scale NUE and PUE data (n = 2,354 for NUE, n = 1,006 for PUE) alongside climate, soil properties, and agricultural management information to delineate the current global spatial distribution patterns of NUE and PUE for these crops and identify their underlying drivers.
Sampling strategy	To quantitatively evaluate global trends and drivers of the NUE and PUE of major crops (rice, wheat, maize, and soybean), we undertook a comprehensive data collection initiative (Supplementary Fig. 7) by a systematic literature review of peer-reviewed articles obtained from the Web of Science (https://www.web of science.com/), Google Scholar (https://scholar.google.com/), and China National Knowledge Infrastructure (https://oversea.cnki.net/index/), up to April 2023 (Supplementary Fig. 7). Articles included in the statistical analysis met the following screening criteria: (1) provided explicit records of NUE and PUE or crop uptake of these nutrients. Both NUE and PUE were calculated based on N(P)UEdiff; (2) spanned at least one complete crop cycle; (3) specify geographical locations; (4) control (no fertilizer) and experimental treatments (fertilizer applied) are executed in the field conditions; and (5) the information on crop species, cropping system, and fertilizer inputs are reported. Since the actual fertilizer use efficiency will not be less than 0, we exclude these outliers. Based on these criteria, our final dataset comprised 2,919 paired independent observations from 173 global studies, including 2,354 N data and 1,006 P data, with sampling years spanning ranging from 1982 to 2021 (Fig. 1).
Data collection	Data extracted from the selected studies included: (1) experimental site details such as latitude, longitude, elevation and the country; (2) crop types; (3) climate conditions, detailed through mean annual precipitation (MAP), mean annual air temperature (MAT), mean annual evapotranspiration (ET), and the aridity index (AI); (4) initial soil physical properties including texture (proportions of sand, silt, and clay) and bulk density (BD), along with soil chemical properties like total carbon (C), nitrogen (N), phosphorus (P), available phosphorus (AP) contents and pH level; (5) the nature of tillage practices, categorized as either conservation or conventional based

on descriptions within the articles; (6) the period of the experiment, indicating the start year and duration; (7) inputs of N, P, and potassium (K) fertilizers; (8) fertilizers application types (FAT) including organic fertilizers, inorganic fertilizers, and the combined application of organic and inorganic fertilizers; (9)fertilizers application placement (FAP) including surface application, deep application, mixed application, and foliar spraying; (10) fertilizer application frequency (FAF); (11) irrigation methods (IM) including permanent flooding, intermittent irrigation, drip irrigation, and no irrigation; (12) the period of the experiment, indicating the start year and duration; (13) the number of experimental replicates conducted; and (14) the NUE and PUE values alongside the N and P uptake by crops. Preference was given to using nutrient uptake data to calculate the efficiencies when both nutrient uptake data and NUE and PUE values were provided. Data were extracted directly from tables and textual descriptions provided in the selected studies. For data presented in graphical form, we employed GetData Graph Digitize software (version 2.25.0.32) to ensure accuracy in data retrieval.

Timing and spatial scale: Time scale is 1961-2018; spatial scale is global cropland

Data exclusions: Articles included in the statistical analysis met the following screening criteria: (1) provided explicit records of NUE and PUE or crop uptake of these nutrients. Both NUE and PUE were calculated based on N(P)UEdiff; (2) spanned at least one complete crop cycle; (3) specify geographical locations; (4) control (no fertilizer) and experimental treatments (fertilizer applied) are executed in the field conditions; and (5) the information on crop species, cropping system, and fertilizer inputs are reported. Since the actual fertilizer use efficiency will not be less than 0, we exclude these outliers. Based on these criteria, our final dataset comprised 2,919 paired independent observations from 173 global studies, including 2,354 N data and 1,006 P data, with sampling years spanning ranging from 1982 to 2021 (Fig. 1).

Reproducibility: The data collection and analysis followed the PRISMA guidelines for reproducibility.

Randomization: The data were analyzed with a random effect model. Details were reported inin Statistical analysis section ooff the Met

Blinding: *Describe the extent of blinding used during data acquisition and analysis. If blinding was not possible, describe why OR explain why blinding was not relevant to your study.*

Did the study involve field work? ☐ Yes ☒ No

Reporting for specific materials, systems and methods

We require information from authors about some types of materials, experimental systems and methods used in many studies. Here, indicate whether each material, system or method listed is relevant to your study. If you are not sure if a list item applies to your research, read the appropriate section before selecting a response.

Materials & experimental systems

n/a	Involved in the study
☒	☐ Antibodies
☒	☐ Eukaryotic cell lines
☒	☐ Palaeontology and archaeology
☒	☐ Animals and other organisms
☒	☐ Clinical data
☒	☐ Dual use research of concern
☒	☐ Plants

Methods

n/a	Involved in the study
☒	☐ ChIP-seq
☒	☐ Flow cytometry
☒	☐ MRI-based neuroimaging

Plants

Seed stocks: *Report on the source of all seed stocks or other plant material used. If applicable, state the seed stock centre and catalogue number. If plant specimens were collected from the field, describe the collection location, date and sampling procedures.*

Novel plant genotypes: *Describe the methods by which all novel plant genotypes were produced. This includes those generated by transgenic approaches, gene editing, chemical/radiation-based mutagenesis and hybridization. For transgenic lines, describe the transformation method, the number of independent lines analyzed and the generation upon which experiments were performed. For gene-edited lines, describe the editor used, the endogenous sequence targeted for editing, the targeting guide RNA sequence (if applicable) and how the editor was applied.*

Authentication: *Describe any authentication procedures for each seed stock used or novel genotype generated. Describe any experiments used to assess the effect of a mutation and, where applicable, how potential secondary effects (e.g. second site T-DNA insertions, mosiacism, off-target gene editing) were examined.*
